# Differences in Levels of Mitochondrial DNA Content at Various Stages of Canine Myxomatous Mitral Valve Disease

**DOI:** 10.3390/ani13243850

**Published:** 2023-12-14

**Authors:** Suphakan Chirathanaphirom, Phongsakorn Chuammitri, Wanpitak Pongkan, Nawin Manachai, Pinkarn Chantawong, Burin Boonsri, Chavalit Boonyapakorn

**Affiliations:** 1 Cardiopulmonary Clinic, Small Animal Hospital, Faculty of Veterinary Medicine, Chiang Mai University, Chiang Mai 50200, Thailand; suphakan_chi@cmu.ac.th (S.C.); wanpitak.p@cmu.ac.th (W.P.); 2 Faculty of Veterinary Medicine, Chiang Mai University, Chiang Mai 50100, Thailand; phongsakorn.c@cmu.ac.th (P.C.); nawin.m@cmu.ac.th (N.M.); pinkarn.chan@cmu.ac.th (P.C.); burin.b@cmu.ac.th (B.B.); 3 Research Center for Veterinary Biosciences and Veterinary Public Health, Chiang Mai 50100, Thailand

**Keywords:** oxidative stress, heart failure, myxomatous mitral valve disease, mitochondrial deoxyribonucleic acid, dogs

## Abstract

**Simple Summary:**

Myxomatous mitral valve disease (MMVD) stands as a prevalent cardiac ailment in the canine population, with the potential to culminate in heart failure. Within the pathogenesis of this condition, oxidative stress assumes a pivotal role, possibly impacting the functionality of the heart’s primary energy production center, the mitochondria. We noticed a gap in research regarding changes in mitochondrial DNA in dogs with this condition. Our study aimed to fill this gap by examining mitochondrial DNA and oxidative stress at different stages of myxomatous mitral valve disease in dogs. Our findings showed that in the early stages of the disease, mitochondrial DNA levels were lower in affected dogs compared to healthy ones. However, mitochondrial DNA levels slightly increased as the disease progressed to the heart failure stage. Additionally, we observed that oxidative stress, a harmful process in the body, increased in dogs with heart disease. Dogs diagnosed with heart failure who received therapeutic interventions exhibited a modest abatement in oxidative stress levels. Our study suggests that in canine myxomatous mitral valve disease, alterations in mitochondrial DNA and elevated oxidative stress manifest in the advanced stages of the condition. These findings shed light on the intricate dynamics of this disease and prompt contemplation regarding the utility of mitochondrial DNA as a potential biomarker for the assessment and prognosis of heart conditions in dogs, thereby illuminating a prospective avenue for further research.

**Abstract:**

Myxomatous mitral valve disease (MMVD) is the most common heart disease in small-breed dogs, often leading to heart failure. Oxidative stress in MMVD can harm mitochondria, decreasing their DNA content. This study assesses dogs’ oxidative stress and mitochondrial DNA at different MMVD stages. Fifty-five small-breed dogs were categorized into four groups, including: A—healthy (*n* = 15); B—subclinical (*n* = 15); C—heart failure (*n* = 15); and D—end-stage MMVD (*n* = 10). Serum malondialdehyde (MDA) and mitochondrial DNA in peripheral blood were analyzed. Quantitative real-time PCR measured mitochondrial DNA, and PCR data were analyzed via the fold-change Ct method. Serum MDA levels were assessed using competitive high-performance liquid chromatography (HPLC). Mitochondrial DNA was significantly lower in group B (−0.89 ± 2.82) than in group A (1.50 ± 2.01), but significantly higher in groups C (2.02 ± 1.44) and D (2.77 ± 1.76) than B. MDA levels were notably elevated in groups B (19.07 ± 11.87 µg/mL), C (23.41 ± 12.87 μg/mL), and D (19.72 ± 16.81 μg/mL) in comparison to group A (9.37 ± 4.67 μg/mL). Nevertheless, this observed difference did not reach statistical significance. It is noteworthy that mitochondrial DNA content experiences a decline during the subclinical stage but undergoes an increase in cases of heart failure. Concurrently, oxidative stress exhibits an upward trend in dogs with MMVD. These findings collectively suggest a potential association between mitochondrial DNA, oxidative stress, and the progression of MMVD in small-breed dogs.

## 1. Introduction

Myxomatous mitral valve disease (MMVD) stands as the predominant acquired cardiac ailment in dogs, particularly prevalent among older small-breed canines [1,2]. The hallmark of myxomatous degeneration manifests in the progressive opacity, thickening, and distortion of the mitral valve, initiating at the valve margin due to the accumulation of extracellular matrix components within the spongiosa layer—specifically glycosaminoglycan and proteoglycan—alongside the disruption of normal collagen alignment in the fibrosa layer [2,3,4]. This degeneration compromises the mitral valve apparatus, resulting in valve insufficiency [3,4]. Consequently, mitral valve regurgitation heightens left atrial pressure, culminating in left atrial dilatation. Elevated left atrial pressure precipitates an increase in pulmonary venous pressure, subsequently inducing the development of pulmonary edema [5]. The American College of Veterinary Internal Medicine (ACVIM) consensus guidelines delineate a comprehensive staging system for MMVD, comprising four distinct stages. Stage A designates a dog at a heightened risk of MMVD development yet devoid of audible murmur heart sounds. Stage B encompasses asymptomatic dogs exhibiting murmur heart sounds, further subdivided into B1, lacking evidence of cardiac remodeling, and B2, manifesting cardiac remodeling necessitating the initiation of pimobendan as a therapeutic intervention. Stage C characterizes dogs showcasing clinical manifestations of heart failure in both present and past contexts, responsive to current treatments encompassing pimobendan, angiotensin-converting enzyme inhibitors (ACEi), and diuretics. Finally, Stage D delineates the end-stage of MMVD, refractory to conventional treatments, mandating a furosemide dosage exceeding 8 mg/kg/day or an equivalent torsemide dose or necessitating the incorporation of anti-arrhythmic drugs [6].

In cases of heart failure, higher levels of oxidative stress have been reported in affected dogs by MMVD [7,8,9]. Oxidative stress results from an imbalance between oxidant and antioxidant agents and is involved in the pathogenesis and progression of heart diseases. Oxidative stress can cause protein modification and lipid peroxidation [10,11], cellular apoptosis [12], endothelial dysfunction [10,13], cardiac remodeling [10,11,12,14], impaired cardiac contraction and function [10,12], and mitochondrial damage and dysfunction [10,11]. Malondialdehyde (MDA) is a secondary product of polyunsaturated fatty acid peroxidation, which is commonly used as an oxidative biomarker in several chronic diseases in clinical studies due to its stability and toxic properties. MDA can irreversibly damage proteins and nucleic acids, resulting in interference with cellular function and the induction of cell death [15,16].

Mitochondria are not only targets of oxidative stress but can themselves be sources of oxidative stress. The primary function of the mitochondria is to produce energy for the myocardium via the oxidative phosphorylation reaction [17,18]. Mitochondria have a genome, which includes circular and unmethylated DNA that is used by transcription proteins in oxidative phosphorylation. The mitochondrial DNA (mtDNA) copy number represents the mitochondrial size and number, which depends on cellular energy demand, oxidative stress, and pathological conditions. The mtDNA is easily damaged by ROS resulting from a lack of histone proteins, chromatin structure, and limited repair activity [18,19]. Mitochondrial damage resulting from oxidative stress is reflected by a decrease in mtDNA copy number [19]. In humans, mtDNA has been reported to be a useful biomarker for mitochondrial function and prognosis in cardiac diseases [18,20,21]. The mtDNA content can be determined by calculating the ratio of the mitochondrially encoded gene to the nuclear-encoded gene (Mt/N) using the real-time PCR technique, the method of choice for the last decade [18,22]. The peripheral blood mtDNA content is useful as an indirect biomarker for evaluating mitochondrial function [22]. mtDNA content can be measured from specific tissue, but doing so is impractical in clinical practice. Decreased peripheral mtDNA has been shown in humans with heart failure [21,23] but has not yet been studied in dogs. The objective of this study is to evaluate mtDNA content, oxidative status, and cardiac function in dogs with different stages of MMVD. We hypothesized that depleted mitochondrial function would occur in MMVD dogs with increased oxidative stress.

## 2. Methods

### 2.1. Animals

The 55 client-owned dogs, which were patients in the cardiology clinic at the Small Animal Hospital, Chiang Mai University, were small breeds, aged more than 5 years, and of both sexes. All dogs were classified into four groups according to the American College of Veterinary Internal Medicine (ACVIM) stages of heart disease and heart failure [6] as follows: group A—healthy and predisposed breed dogs (*n* = 15), group B—subclinical stage (*n* = 15), group C—heart failure stage (*n* = 15), and group D—end-stage (*n* = 10). All animals had been clinically stable for at least 1 month before blood sample collection. Dogs with infections, inflammation, or other systemic diseases, such as neoplasia, urinary disease, hormonal disease, neurologic disorder, and hepatic disease, were excluded from the study. The study protocol was approved by the Ethics Committee of the Laboratory Animal Center, Faculty of Veterinary Medicine, Chiang Mai University (S29/2564), and all owners signed an informed consent form.

### 2.2. Physical Examinations

A complete physical examination was performed on all recruited dogs. Clinical data collected included body weight, body temperature, and vital signs. The heart sound was recorded as either normal or a murmur and was categorized into six grades [24].

### 2.3. Thoracic Radiography

Right lateral and dorsoventral thoracic radiography was performed to measure the heart size. The vertebral heart size (VHS) [25] and the vertebral left atrial size (VLAS) [26] were measured in the right lateral position.

### 2.4. Echocardiography

Echocardiography was used to evaluate cardiac structure and function. Non-sedated dogs were placed in the right lateral position for the right parasternal long-axis four-chamber view for evaluating mitral valve structure and in the right parasternal short-axis view at the aortic root for evaluating left atrial dimension (LA/Ao) using the Swedish method [27]. The 2D-guided M-mode was used to determine the left ventricular dimensions. The dogs were then placed in the left lateral position to observe the mitral valve structure and regurgitation jet and to measure left ventricular function. The severity of mitral valve regurgitation was assessed by the semi-quantification method using the color-flow Doppler mode to evaluate the maximal ratio of the regurgitant jet area (ARJ) to the left atrial area (LAA). The severity of mitral valve regurgitation was considered as mild, moderate, and severe when the ARJ/LAA ratio was less than 20–30%, 30–70%, and more than 70%, respectively [28]. The diastolic and systolic ventricular volume and ejection fraction were measured by the modified Simpson’s method in the left apical four-chamber view [29].

### 2.5. Blood Sample Collection

Three milliliters (mL) of blood were collected from the cephalic or saphenous vein and were placed into 1 mL in EDTA tubes for hematology and mitochondrial DNA content, 1 mL in a plain tube for MDA analysis, and 1 mL in a heparin tube for biochemistry profile. The hematology and biochemistry profiles were measured immediately after collection. The serum was separated by centrifugation at 3500× *g* for 5 min. The rest of the EDTA blood and serum was kept at −20 °C for later mitochondrial DNA extraction and MDA analysis.

### 2.6. Mitochondrial DNA Extraction and Determination of Mitochondrial DNA Content

EDTA blood samples were thawed at room temperature. The mitochondrial DNA was extracted from the whole blood using a NucleoSpin^®^ Tissue kit (Macherey-Nagel, Düren, Germany), following the manufacturer’s instructions. Real-time PCR was used to determine peripheral blood mitochondrial DNA content. The mitochondrial-encoded NADH-ubiquinone oxidoreductase subunit 4 (Mt-ND4) was amplified as a mitochondrial gene. The forward and reverse mitochondrial primer sequences were 5′-CGTAATCAGTCCCGTAGGTGTTAGA-3′ and 5′-ACATTAGCCAGCATGATACCAATCG-3′ [30]. Glyceraldehyde-3-phosphate dehydrogenase (GAPDH) was used as a housekeeping gene. The forward and reverse primers of GAPDH were designed as 5′-TCACCAGGGCTGCTTTTAAC-3′ and 5′-TGACTGTGCCGTGGAATTTG-3′. The real-time PCR was run using the HOT FIREPol^®^ EvaGreen^®^ qPCR Mix Plus (ROX) (Solis BioDyne, Tartu, Estonia). The annealing temperature for both genes was set at 60 °C. All samples were run in duplicate using ABI Prism 7300 real-time PCR (Applied Biosystems, Thermo Fisher Scientific, MA, USA). A cycle threshold (Ct) value of mitochondrial genes and housekeeping genes was calculated for each group. mtDNA levels were normalized using GAPDH as an endogenous control and were calculated using the 2^−∆Ct^ method [31].

### 2.7. Determination of Malondialdehyde Concentration

Serum samples were thawed at room temperature. MDA concentration was analyzed using a competitive HPLC technique [32]. The serum sample was mixed with 10% trichloroacetic acid and incubated at 90 °C for 30 min. After cooling to room temperature, the mixture was centrifuged at 6000× *g* rpm for 10 min. The supernatant was collected and mixed with 0.44M phosphoric acid (H_3_PO_4_) and 0.6% thiobarbituric acid (TBA) to generate thiobarbituric acid reactive substances (TBARS), a pink-colored product, after being incubated at 90 °C for 30 min. The solution was filtered using a syringe filter (nylon membrane, pore size 0.45 µm, HAMAG^®^, Ningbo, China) and then analyzed with an HPLC system. The mobile phase consisted of potassium phosphate buffer and methanol in a ratio of 1:1. The flow rate of the mobile phase was 1.0 mL/min. The fluorescent color was detected at 532 nm. The temperature of the column oven was set at 35 °C. The standard curve was created from a standard reagent of malondialdehyde at different concentrations (0–100 µM). The TBARS concentration was determined directly from a standard curve and reported as MDA equivalent concentration in µM [33].

### 2.8. Statistical Analysis

For statistical analysis, IBM SPSS Statistics 21.0 was used. The normal distribution of data was tested using the Shapiro−Wilk test. Statistical significance was analyzed using the one-way ANOVA for parametric statistics and the Kruskal-Wallis test for nonparametric statistics. The Duncan test was used for pairwise multiple comparisons. Statistical significance was set at a *p*-value < 0.05. The data are presented as mean ± SD.

## 3. Results

### 3.1. Baseline Data

The breeds of dogs in the study included Chihuahua (*n* = 19), Pomeranian (*n* = 16), Shih Tzu (*n* = 9), Poodle (*n* = 8), Schnauzer (*n* = 1), and mixed breed (*n* = 2). There were 29 males and 26 females among the 55 dogs. The average age was 10.2 ± 2.7 years old. The average body weight and body temperature were 4.8 ± 2.1 kg and 101.0 ± 0.4 °F, respectively. The average heart and respiratory rates were 124 ± 16.3 beats per minute and 36.2 ± 10.8 breaths per minute, respectively. One dog in Group D had atrial fibrillation. Except for group A, which had a normal heart sound, all of the heart sounds in the other groups were murmurs of varying severity. There was no significant difference in clinical data within any of the groups. Clinical data for the different groups are shown in Table 1.

The MMVD-afflicted dogs in this study underwent treatment in accordance with the guidelines established by the American College of Veterinary Internal Medicine (ACVIM), as outlined in Table 2. Within Group B, eight out of fifteen dogs received pimobendan. Group C comprised dogs that all received pimobendan and furosemide, along with additional pharmacological interventions, including spironolactone (6/15), ramipril (4/15), benazepril (8/15), and sildenafil (3/15). In Group D, consisting of end-stage MMVD cases, ten out of ten dogs were administered furosemide and pimobendan. Additionally, they were prescribed a combination of other medications, namely spironolactone (9/10), ramipril (4/10), benazepril (4/10), sildenafil (4/10), and diltiazem (1/10).

### 3.2. Hematology and Blood Chemistry Profiles

For the hematology profiles, there were no significant differences in any hematology parameters, as shown in Table 3. Besides the blood chemistry profile, the creatinine and blood urea nitrogen (BUN) levels were significantly higher in group D than in groups A, B, and C, as shown in Table 3 (*p* < 0.001).

### 3.3. Thoracic Radiographic Characteristics

Thoracic radiography showed VHS was significantly higher in group D than in groups A, B, and C. The VHS was significantly higher in groups C than in groups A and B (*p* < 0.001). The VLAS was significantly higher in groups C and D when compared with groups A and B (*p* < 0.001, Table 4).

### 3.4. Echocardiographic Characteristics

The echocardiographic parameters of LA/Ao did not exhibit statistically significant differences between groups A and B; however, the LA/Ao was markedly higher in both groups C and D compared to groups A and B (*p* < 0.001). Left ventricular diameter in diastole (LVIDD) demonstrated a significant increase in group D in comparison to groups A, B, and C, and LVIDD in group C was also significantly higher than that in groups A and B (*p* < 0.001). The normalized left ventricular internal diameter in diastole (LVIDDN) exhibited statistically significant elevation in groups C and D when compared with groups A and B (*p* < 0.001). Furthermore, the left ventricular internal diameter in systole (LVIDS) and left ventricular posterior wall in systole were both significantly higher in group D as opposed to groups A and B (*p* = 0.02 and *p* = 0.02, respectively).

Regarding systolic function, there were no significant differences observed in ejection fraction percentages between the groups. However, the percentage of fractional shortening exhibited a significant increase in group C compared to group A (*p* = 0.02). Mitral valve regurgitation was detected in groups B, C, and D, with the severity varying among the groups, while no mitral valve regurgitation was observed in group A. End-diastolic volume (EDV) was significantly higher in groups C and D compared to groups A and B (*p* < 0.001). Stroke volume (SV) showed a significant increase in group D when compared to groups A, B, and C, and it was also significantly higher in group C than in groups A and B (*p* < 0.001).

### 3.5. Mitochondrial DNA Content

**Figure 1:**
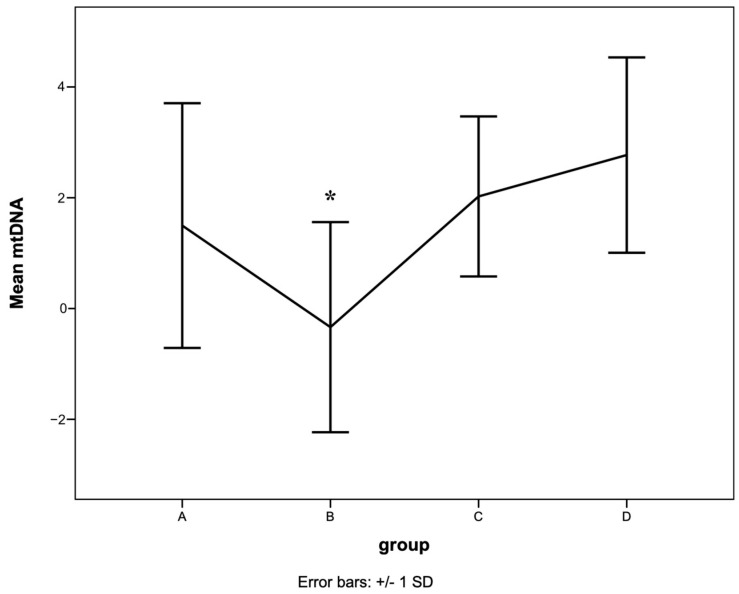
The mean mtDNA content in dogs in different MMVD groups (A, B, C, and D). The line graph displays the mean and standard deviation of mtDNA content in different groups of MMVD dogs. The mtDNA content decreased in group B significantly when compared to the other groups. * represents statistical significance.

The mean mtDNA content was significantly lower in group B (mtDNA = −0.3 ± 1.9, *p* = 0.001, Figure 1) compared with group A (mtDNA = 1.5 ± 2.2). In contrast, mtDNA content was significantly higher in groups C (mtDNA = 2.0 ± 1.4) and D (mtDNA = 2.8 ± 1.8) compared with group B.

### 3.6. Malondialdehyde Level

**Figure 2:**
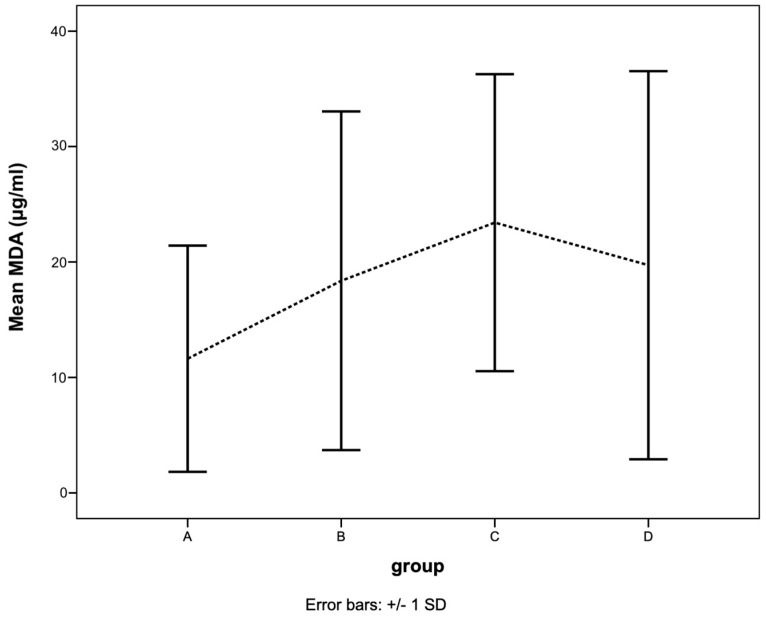
The mean of MDA level in dogs in different MMVD groups (A, B, C, and D). The line shows the mean with standard deviation of MDA level in different groups of MMVD dogs. The MDA level was high in groups B, C, and D but did not show statistical significance.

The mean malondialdehyde (MDA) levels in MMVD dogs (group B: 18.4 ± 14.7 µg/mL, group C: 23.4 ± 12.9 µg/mL, group D: 19.7 ± 16.8 µg/mL) were higher than those in healthy dogs (group A: 11.6 ± 9.8 µg/mL), but there was no statistical difference (Figure 2).

## 4. Discussion

The main finding of this study was that the mtDNA content changed during the MMVD stages. Notably, mtDNA content exhibited a marked reduction during the subclinical stage (group B) but conversely presented heightened levels in the clinical stages associated with heart failure (groups C and D). Within group B, it is evident that the mtDNA content exhibited a statistically significant decrease when compared to the healthy dogs in group A. Conversely, in groups C and D, the mtDNA content displayed a statistically significant increase when compared to group B. The concentration of MDA, a biomarker for oxidative stress, was elevated in dogs afflicted by MMVD. However, it did not exhibit a statistically significant difference when compared to the MDA levels in healthy dogs (group A). These findings emphasize that both MDA levels and mtDNA content in MMVD dogs undergo nonuniform changes at distinct stages of the disease.

The mtDNA content originates from two primary sources: plasma mtDNA and peripheral white blood cell mtDNA. Plasma mtDNA is released from damaged cells, such as cardiomyocytes, white blood cells, and other cells, as a response to oxidative stress [34,35]. Our study’s results indicate a decrease in mtDNA content in group B, reflecting mitochondrial dysfunction in the myocardium. This aligns with a study by Knez et al. (2017), who investigated the correlation between myocardial and peripheral blood mtDNA in human patients diagnosed with dilated cardiomyopathy (DCM) after heart transplantation [23]. Their findings provided evidence of alterations in mtDNA within the myocardium, suggesting a potential correlation between myocardial and peripheral blood mtDNA content [23].

Studies investigating chronic heart failure patients have produced intriguing results regarding mtDNA content in peripheral white blood cells. These investigations have consistently reported a reduction in peripheral mtDNA content [21]. Importantly, this decline in peripheral mtDNA content has shown a significant correlation with an elevated risk of heart failure, increased occurrences of cardiovascular rehospitalization, and higher mortality rates [20,21]. The notable reduction in mtDNA content observed in group B in our study holds particular significance, indicating that dogs in this group may be at a heightened risk of developing heart failure compared to the healthy dogs in group A. This observation aligns with existing research, highlighting the connection between diminished mitochondrial function in the context of cardiovascular disease and its involvement in processes such as oxidative stress-induced cardiac remodeling and cardiomyocyte death [21]. Consistently, a recent study involving middle-aged women has unveiled a compelling connection between a lower mtDNA copy number obtained from whole blood and an increased incidence of heart failure, alongside an elevated risk of heart failure-related mortality due to myocardial infarction [36].

On the other hand, in dogs with heart failure, there is an increase in mtDNA content due to the release of mtDNA from damaged cells. This observation is supported by the study conducted by Dhondup et al. (2016), which observed increased plasma mtDNA content in chronic heart failure patients compared to healthy patients [34]. Importantly, Dhondup et al. found that this increased plasma mtDNA content was not significantly correlated with the severity of heart failure. The elevation in plasma mtDNA content observed in heart failure cases may result from increased mtDNA release or degradation from failing myocardial tissue, alongside other cells or organs, due to prolonged cardiac stress [34].

Moreover, a 2020 study by Krychtiuk and colleagues demonstrated a noteworthy increase in plasma mtDNA content corresponding to higher New York Heart Association (NYHA) (New York, NY, USA) classes of heart failure. The highest levels of plasma mtDNA were observed in cases of acute heart failure. Their hypothesis suggests that factors such as hypoperfusion and cell death in heart failure contribute to mtDNA release, positioning mtDNA as a potential biomarker for generalized, nonorgan-specific hypoperfusion and cell death [35]. Another investigation involving acute myocardial infarction patients and isolated perfused murine hearts revealed a direct correlation between the release of mtDNA into plasma and the extent of myocardial damage, further supporting the role of mtDNA as a valuable indicator of cardiac stress and damage [37].

A study by Anguita et al. (2022) indicated that mtDNA content derived from peripheral blood mononuclear cells (PBMC) in heart failure patients with preserved function displayed an increase during the chronic stage. However, intriguingly, there was no significant change in mitochondrial mass when compared with age- and sex-matched control subjects [38]. The study that observed an increase in mtDNA content in the chronic stage of heart failure suggested that this rise could be attributed to the chronic high energy demand faced by the cardiovascular system [38]. This hypothesis aligns with the notion that in cases of preserved cardiac function during heart failure, such as in MMVD, the increase in mtDNA content may be a compensatory mechanism deployed by the cardiovascular system to address the persistent energy requirements imposed by the condition. Moreover, a recent study conducted using a rat model of mitral regurgitation demonstrated the effectiveness of pimobendan in preserving the structural integrity of cardiac mitochondria. The notable increase in mtDNA content observed in groups C and D, in contrast to group B, could reasonably be ascribed to the therapeutic impacts of the medication administered for the management of heart failure [39].

The mtDNA is known to elicit sterile inflammation through two distinct pathways. Firstly, mtDNA can trigger the innate immune system, inciting sterile inflammation by activating toll-like receptor 9 (TLR9) [40,41,42]. Secondly, mtDNA stimulates the nucleotide-binding oligomerization domain (NLRP3), subsequently leading to the release of pro-inflammatory cytokines [42,43]. The escape of mtDNA-induced sterile inflammation can lead to myocarditis and fibrosis [40,44]. This theory was confirmed in a study in DNase II-deficient mice induced to develop hypertrophic cardiomyopathy and heart failure by transverse aortic constriction (TAC), which showed accumulation of mtDNA in autolysosomes, infiltration of inflammatory cells in the myocardium, and increased RNA expression of inflammatory cytokines after TAC. Inhibition of TLR9 also attenuated the development of cardiomyopathy [40]. Additionally, a recent investigation involving patients who underwent cardiac surgery unveiled an elevation in mtDNA levels among patients who experienced postsurgical complications and developed multiple organ failure (MOF) [43]. This empirical evidence further reinforces the theory that mtDNA plays a pivotal role in activating sterile inflammatory responses, potentially resulting in the onset of systemic inflammatory response syndrome (SIRS) and multiple organ failure [35,43]. Although our study did not encompass comprehensive data on specific inflammatory biomarkers, it is noteworthy that we observed a slight elevation in the white blood cell count in dogs afflicted by heart failure. This increase may be indicative of an inflammatory response activated by plasma mtDNA, a phenomenon paralleling observation in studies of chronic heart failure patients [34] and investigations involving dogs with MMVD [8,45,46]. These collective findings shed light on the intricate interplay between mtDNA and inflammation in the context of cardiac diseases.

In our current investigation, we determined that the level of MDA did not exhibit an association with the stage of MMVD. This outcome parallels findings from prior research in dogs with heart failure [47,48]. Furthermore, earlier investigations into canine MMVD and DCM have reported a lack of statistically significant associations between MDA levels in dogs at various stages of MMVD and healthy control subjects [9,48]. Likewise, another investigation involving dogs with MMVD reported a lack of correlation between MDA levels and the stage of the disease. Interestingly, the study did reveal an increase in MDA levels in the untreated heart failure group when compared to the group receiving treatment for heart failure. The authors of this study suggest that the absence of a correlation between MDA levels and the stage of MMVD may be attributed to the impact of treatment during the heart failure phase [47]. In accordance with our findings, we observed that the level of MDA was lower in stage D when compared to stage B. This reduction in MDA levels may be attributed to the potential antioxidant effects of medications used to manage heart failure during stage D, such as benazepril [49] and sildenafil [50]. Furthermore, a prior study conducted in chronic volume overload dogs indicated that an elevation in MDA levels within cardiac tissue may not necessarily correlate with MDA levels in plasma. This finding suggests that there might be no direct association between MDA levels and the stage of heart disease [47]. Numerous studies on human patients with various heart conditions, including DCM, ischemic and nonischemic heart diseases, and chronic heart failure, have consistently reported higher concentrations of MDA in individuals afflicted by heart disease and heart failure [51,52,53]. It is important to note, however, that the precise relationship between MDA concentration and the severity of heart disease has not been conclusively established. Indeed, the relationship between MDA levels and various parameters in heart disease patients can be complex and, at times, inconclusive. For instance, one study involving chronic heart failure patients discovered a negative correlation between MDA levels and left ventricular ejection fraction [54]. In contrast, another study conducted with dilated cardiomyopathy (DCM) patients found no significant relationship between MDA levels and the New York Heart Association (NYHA) class, echocardiographic parameters, or hemodynamic parameters [53].

The changes observed in both mtDNA content and MDA levels did not conform to the hypothesis that oxidative stress induces damage to mtDNA, ultimately resulting in reduced mtDNA content and subsequent mitochondrial dysfunction [18,19]. Although there may be no prior study explicitly demonstrating a direct relationship between oxidative stress biomarkers and mtDNA, there is existing research exploring the broader connection between oxidative stress and DNA damage in cardiovascular patients. Specifically, studies conducted by Qasim et al. and Bhat and Gandhi both reported a negative correlation between oxidative DNA damage and total antioxidant status [55,56]. These studies lend support to the hypothesis that oxidative stress can lead to mtDNA damage in the context of cardiovascular diseases.

To the best of our knowledge, this is the first study to reveal variations in peripheral blood mtDNA content among dogs at different stages of MMVD. Our findings may introduce malondialdehyde (MDA) and mitochondrial DNA (mtDNA) as novel biomarkers for prognosticating the progression of myxomatous mitral valve disease (MMVD). However, to enhance accuracy, a larger sample size is warranted. Unfortunately, as of now, laboratories equipped for MDA analysis and mtDNA content assessment are not available in routine clinical practice. The present study is subject to several limitations. Firstly, the sample size included multiple breeds, and there was a lack of breed-, age-, sex-, and neuter status-matched controls, which could potentially introduce variations in mtDNA content and MDA levels. In our comprehensive analysis of blood profiles, it is imperative to acknowledge a notable omission in the assessment of electrolyte and phosphorus levels among dogs undergoing treatment for heart failure. This absence of data pertaining to electrolyte and phosphorus concentrations is particularly significant in the comprehensive evaluation of the disease status. Subsequent incorporation of these parameters will contribute valuable insights to the nuanced evaluation of the therapeutic outcomes and overall well-being of the subjects undergoing heart failure management. Furthermore, our cross-sectional study lacks serial data on blood profiles, mtDNA content, and MDA levels throughout the progression of the disease within the same dog. It is also important to note that the mtDNA was extracted from whole blood, potentially containing mtDNA from both peripheral white blood cells and free-floating mtDNA in plasma. This possibility of different origins of mtDNA raises the potential for diverse pathogeneses being involved in the development of oxidative stress and heart failure. For future research endeavors, it would be valuable to conduct separate analyses of plasma and peripheral white blood mtDNA as they may exhibit distinct patterns of change. Additionally, it would be beneficial to explore mitochondrial mass and dynamics, as well as investigate potential associations between mtDNA content and inflammatory biomarkers. These supplementary investigations could provide a more comprehensive understanding of the interplay between mtDNA, oxidative stress, and heart disease.

## 5. Conclusions

The study documented a reduction in mtDNA content during the subclinical stage, followed by an increase in the heart failure stage in dogs with MMVD. While this study did not definitively establish a direct association between mtDNA content, MMVD stages, or oxidative status, it does serve as the pioneering investigation into the alterations in peripheral blood mitochondrial DNA content across the various stages of MMVD in dogs.

## Figures and Tables

**Table 1:** Clinical data of dogs by group. Age, body weight, body temperature, heart rate, and respiratory rate are presented as mean ± SD. Sex and heart sound are presented as the number of subjects.

Group (MMVD Stage)	A (*n* = 15)	B (*n* = 15)	C (*n* = 15)	D (*n* = 10)	*p*-Value
Age (years)	8.9 ± 3.1	10.5 ± 2.4	10.9 ± 2.4	10.8 ± 2.9	0.17
Sex (male/female)	6/9	8/7	9/6	6/4	N/A
Body weight (kg)	4.4 ± 1.9	4.9 ± 2.0	4.6 ± 3.2	5.9 ± 3.2	0.38
Heart rate (bpm)	130 ± 17.7	124.0 ± 15.9	121.2 ± 12.4	123.0 ± 20.0	0.50
Heart murmur (0/I/II/III/IV/V/VI)	15/0/0/0/0/0/0	0/0/2/5/6/2/0	0/0/0/0/7/8/0	0/0/0/0/1/9/0	N/A
Respiratory rate (BPM)	37.9 ± 10.8	33.6 ± 12.2	35.2 ± 8.3	37.3 ± 10.6	0.52

N/A, not applicable.

**Table 2:** Pharmacological treatment of dogs by group. The data are presented as the number of subjects.

Group (MMVD Stage)	A (*n* = 15)	B (*n* = 15)	C (*n* = 15)	D (*n* = 10)
Pimobendan	0/15	8/15	15/15	10/10
Furosemide	0/15	0/15	15/15	10/10
Spironolactone	0/15	0/15	6/15	9/10
Ramipril	0/15	0/15	4/15	4/10
Benazepril	0/15	0/15	8/15	4/10
Sildenafil	0/15	0/15	3/15	4/10
Diltiazem	0/15	0/15	0/15	1/10

**Table 3:** The hematology and blood chemistry profiles of dogs in each group. Values are presented as mean ± SD.

Group (MMVD Stage)	A (*n* = 15)	B (*n* = 15)	C (*n* = 15)	D (*n* = 10)	*p*-Value
Hematology parameters					
Hct (%)	50.9 ± 6.2	49.2 ± 5.2	50.2 ± 9.6	51.2 ± 12.2	0.94
RBC (×10^6^/uL)	7.5 ± 0.9	7.0 ± 0.7	7.3 ± 1.5	7.5 ± 1.3	0.57
WBC (×10^3^/uL)	10.6 ± 3.7	10.5 ± 3.1	13.9 ± 5.1	12.4 ± 3.6	0.07
Neutrophil (×10^3^/uL)	7.1 ± 2.9	6.6 ± 2.7	8.9 ± 4.9	9.0 ± 3.0	0.21
Lymphocyte (×10^3^/uL)	2.2 ± 1.0	2.6 ± 1.0	2.9 ± 2.4	1.9 ± 0.6	0.41
Monocyte (×10^3^/uL)	0.6 ± 0.3	0.6 ± 0.4	1.0 ± 0.5	0.8 ± 0.6	0.17
Eosinophil (×10^3^/uL)	0.6 ± 0.4	0.5 ± 0.4	0.5 ± 0.3	0.7 ± 0.3	0.75
Basophil (×10^3^/uL)	0.01 ± 0.02	0.01 ± 0.02	0.02 ± 0.04	0.01 ± 0.01	0.70
Blood chemistry parameters					
Creatinine (mg/dL)	0.9 ± 0.2 ^b^	1.0 ± 0.3 ^b^	1.1 ± 0.3 ^b^	1.8 ± 0.5 ^a^	<0.001
BUN (mg/dL)	21.02 ± 10.62 ^b^	19.11 ± 7.38 ^b^	27.07 ± 13.09 ^b^	57.02 ± 25.37 ^a^	<0.001
ALT (U/L)	62.5 ± 20.0	65.1 ± 61.7	79.5 ± 47.8	66.4 ± 62.0	0.79

HCT, hematocrit; RBC, red blood cell count; WBC, white blood cell count; BUN, blood urea nitrogen; ALT, alanine transaminase. In the same row, the statistical significance showed with different superscripts.

**Table 4:** Thoracic radiographic and echocardiographic characteristics of dogs by group. Values are presented as mean ± SD, except the severity of MR, which is presented as the number of subjects.

Group (MMVD Stage)	A (*n* = 15)	B (*n* = 15)	C (*n* = 15)	D (*n* = 10)	*p*-Value
Radiographic parameters					
VHS	9.9 ± 0.8 ^c^	10.4 ± 1.0 ^c^	11.9 ± 0.8 ^b^	12.8 ± 1.3 ^a^	<0.001
VLAS	1.9 ± 0.2 ^b^	2.1 ± 0.5 ^b^	2.8 ± 0.6 ^a^	3.0 ± 0.7 ^a^	<0.001
Echocardiography parameters					
LA/Ao	1.4 ± 0.1 ^c^	1.7 ± 0.3 ^b^	2.3 ± 0.4 ^a^	2.3 ± 0.5 ^a^	<0.001
IVSD (mm)	0.7 ± 0.1	0.6 ± 0.1	0.6 ± 0.2	0.6 ± 0.1	0.33
LVIDD (mm)	1.9 ± 0.4 ^c^	2.4 ± 0.5 ^c^	3.0 ± 0.5 ^b^	3.5 ± 1.1 ^a^	<0.001
LVIDDn (mm)	1.3 ± 0.2 ^c^	1.6 ± 0.3 ^b^	1.9 ± 0.3 ^a^	1.9 ± 0.4 ^a^	<0.001
LVPWD (mm)	0.6 ± 0.1	0.6 ± 0.1	0.6 ± 0.1	0.7 ± 0.1	0.17
IVSS (mm)	0.9 ± 0.1	0.9 ± 0.2	1.0 ± 0.2	1.1 ± 0.3	0.07
LVPWS (mm)	0.9 ± 0.2 ^b^	1.0 ± 0.2 ^b^	1.0 ± 0.2 ^a,b^	1.2 ± 0.2 ^a^	0.02
FS (MM-Teich %)	43.1 ± 11.9 ^b^	50.6 ± 7.9 ^a,b^	53.9 ± 7.0 ^a^	51.5 ± 6.0 ^a,b^	0.02
Severity of MR (mild/moderate/severe)	N/A	2/4/9	0/4/11	0/0/10	N/A
EDV (A4C, mL)	6.0 ± 2.7 ^b^	7.7 ± 5.0 ^b^	13.6 ± 6.6 ^a^	18.4 ± 11.5 ^a^	<0.001
ESV (A4C, mL)	1.4 ± 1.1	1.6 ± 1.1	1.9 ± 1.0	2.3 ± 2.0	0.12
EF (A4C, %)	76.7 ± 13.3	82.5 ± 8.3	82.9 ± 7.9	86.3 ± 5.8	0.09
SV (A4C, mL)	4.6 ± 2.0 ^c^	6.1 ± 4.2 ^c^	11.7 ± 6.2 ^b^	16.1 ± 9.7 ^a^	<0.001

VHS, vertebral heart scale; VLAS, vertebral left atrial size; LA/Ao, left atrium per aortic root ratio; IVSD, interventricular septum in diastole; LVIDD, left ventricle internal diameter in diastole; LVIDDN, normalized left ventricle internal diameter in diastole; LVPWD, left ventricular posterior wall in diastole; IVSS, interventricular septum in systole; LVIDS, left ventricle internal diameter in systole; LVPWS, left ventricular posterior wall in systole; FS, fractional shortening; EF, ejection fraction; MR, mitral valve regurgitation; EDV, end-diastolic volume; ESV, end-systolic volume; SV, stroke volume; N/A, not applicable. In the same row, the statistical significance is indicated with different superscripts.

## Data Availability

Data are contained within the article.

## References

[1] Mattin M., Boswood A., Church D., López-Alvarez J., McGreevy P., O’Neill D., Thomson P., Brodbelt D. (2015). Prevalence of and risk factors for degenerative mitral valve disease in dogs attending primary-care veterinary practices in England. J. Vet. Intern. Med..

[2] Orozco S.C., Olivera-Angel M., Vargas-Pinto P. (2019). Insights on the canine mitral valve in the course of myxomatous mitral valve disease. CES Med. Vet. Y Zoo..

[3] Borgarelli M., Buchanan J.W. (2012). Historical review, epidemiology and natural history of degenerative mitral valve disease. J. Vet. Cardiol..

[4] Fox P.R. (2012). Pathology of myxomatous mitral valve disease in the dog. J. Vet. Cardiol..

[5] Tilley L.P. (2008). Manual of Canine and Feline Cardiology.

[6] Keene B.W., Atkins C.E., Bonagura J.D., Fox P.R., Häggström J., Fuentes V.L., Oyama M.A., Rush J.E., Stepien R., Uechi M. (2019). ACVIM consensus guidelines for the diagnosis and treatment of myxomatous mitral valve disease in dogs. J. Vet. Intern. Med..

[7] Michałek M., Tabiś A., Cepiel A., Noszczyk-Nowak A. (2020). Antioxidative enzyme activity and total antioxidant capacity in serum of dogs with degenerative mitral valve disease. Can. J. Vet. Res..

[8] Rubio C.P., Saril A., Kocaturk M., Tanaka R., Koch J., Ceron J.J., Yilmaz Z. (2020). Changes of inflammatory and oxidative stress biomarkers in dogs with different stages of heart failure. BMC Vet. Res..

[9] Svete A.N., Verk B., Čebulj-Kadunc N., Salobir J., Rezar V., Petrič A.D. (2021). Inflammation and its association with oxidative stress in dogs with heart failure. BMC Vet. Res..

[10] Kiyuna L.A., Albuquerque R.P.E., Chen C.H., Mochly-Rosen D., Ferreira J.C.B. (2018). Targeting mitochondrial dysfunction and oxidative stress in heart failure: Challenges and opportunities. Free Radic. Biol. Med..

[11] Okonko D.O., Shah A.M. (2015). Heart failure: Mitochondrial dysfunction and oxidative stress in CHF. Nat. Rev. Cardiol..

[12] Tsutsui H., Kinugawa S., Matsushima S. (2011). Oxidative stress and heart failure. Am. J. Physiol. Heart Circ. Physiol..

[13] Zuchi C., Tritto I., Carluccio E., Mattei C., Cattadori G., Ambrosio G. (2020). Role of endothelial dysfunction in heart failure. Heart Fail. Rev..

[14] Sabri A., Hughie H.H., Lucchesi P.A. (2003). Regulation of hypertrophic and apoptotic signaling pathways by reactive oxygen species in cardiac myocytes. Antioxid. Redox Signal..

[15] Del Rio D., Stewart A.J., Pellegrini N. (2005). A review of recent studies on malondialdehyde as toxic molecule and biological marker of oxidative stress. Nutr. Metab. Cardiovasc. Dis..

[16] Ayala A., Muñoz M.F., Argüelles S. (2014). Lipid peroxidation: Production, metabolism, and signaling mechanisms of malondialdehyde and 4-hydroxy-2-nonenal. Oxidative Med. Cell. Longev..

[17] Zhou B., Tian R. (2018). Mitochondrial dysfunction in pathophysiology of heart failure. J. Clin. Investig..

[18] Malik A.N., Czajka A. (2013). Is mitochondrial DNA content a potential biomarker of mitochondrial dysfunction?. Mitochondrion.

[19] Tsutsui H., Kinugawa S., Matsushima S. (2008). Oxidative stress and mitochondrial DNA damage in heart failure. Circ. J..

[20] Hong Y.S., Longchamps R.J., Zhao D., Castellani C.A., Loehr L.R., Chang P.P., Matsushita K., Grove M.L., Boerwinkle E., Arking D.E. (2020). Mitochondrial DNA copy number and incident heart failure: The Atherosclerosis Risk in Communities (ARIC) study. Circulation.

[21] Huang J., Tan L., Shen R., Zhang L., Zuo H., Wang D.W. (2016). Decreased peripheral mitochondrial DNA copy number is associated with the risk of heart failure and long-term outcomes. Medicine.

[22] Kuznetsova T., Knez J. (2017). Peripheral blood mitochondrial DNA and myocardial function. Adv. Exp. Med. Biol..

[23] Knez J., Lakota K., Božič N., Okrajšek R., Cauwenberghs N., Thijs L., Kneževič I., Vrtovec B., Tomšič M., Čučnik S. (2017). Correlation between mitochondrial DNA content measured in myocardium and peripheral blood of patients with non-ischemic heart failure. Genet. Test. Mol. Biomark..

[24] Rishniw M. (2018). Murmur grading in humans and animals: Past and present. J. Vet. Cardiol..

[25] Gugjoo M.B., Hoque M., Saxena A.C., Zama M.M.S., Amarpal A. (2013). Vertebral scale system to measure heart size in dogs in thoracic radiographs. Adv. Anim. Vet. Sci..

[26] Lam C., Gavaghan B.J., Meyers F.E. (2021). Radiographic quantification of left atrial size in dogs with myxomatous mitral valve disease. J. Vet. Intern. Med..

[27] Boon J.A. (2016). Two-Dimensional and M-Mode Echocardiography for the Small Animal Practitioner.

[28] Chetboul V., Tissier R. (2012). Echocardiographic assessment of canine degenerative mitral valve disease. J. Vet. Cardiol..

[29] Boon J.A. (2011). Veterinary Echocardiography.

[30] Appleyard G.D., Forsyth G.W., Kiehlbauch L.M., Sigfrid K.N., Hanik H.L., Quon A., Loewen M.E., Grahn B.H. (2006). Differential mitochondrial DNA and gene expression in inherited retinal dysplasia in miniature Schnauzer dogs. Investig. Ophthalmol. Vis. Sci..

[31] Schmittgen T.D., Livak K.J. (2008). Analyzing real-time PCR data by the comparative C(T) method. Nat. Protoc..

[32] Mateos R., Lecumberri E., Ramos S., Goya L., Bravo L. (2005). Determination of malondialdehyde (MDA) by high-performance liquid chromatography in serum and liver as a biomarker for oxidative stress. Application to a rat model for hypercholesterolemia and evaluation of the effect of diets rich in phenolic antioxidants from fruits. J. Chromatogr. B Anal. Technol. Biomed. Life Sci..

[33] Chueainta P., Punyapornwithaya V., Tangjitjaroen W., Pongkan W., Boonyapakorn C. (2022). Acupuncture Improves Heart Rate Variability, Oxidative Stress Level, Exercise Tolerance, and Quality of Life in Tracheal Collapse Dogs. Vet. Sci..

[34] Dhondup Y., Ueland T., Dahl C.P., Askevold E.T., Sandanger Ø., Fiane A., Ohm I.K., Sjaastad I., Finsen A.V., Wæhre A. (2016). Low circulating levels of mitochondrial and high levels of nuclear DNA predict mortality in chronic heart failure. J. Card. Fail..

[35] Krychtiuk K.A., Wurm R., Ruhittel S., Lenz M., Huber K., Wojta J., Heinz G., Hülsmann M., Speidl W.S. (2020). Release of mitochondrial DNA is associated with mortality in severe acute heart failure. Eur. Heart J. Acute Cardiovasc. Care.

[36] Sundquist K., Sundquist J., Wang X., Palmer K., Memon A.A. (2022). Baseline mitochondrial DNA copy number and heart failure incidence and its role in overall and heart failure mortality in middle-aged women. Front. Cardiovasc. Med..

[37] Bliksøen M., Mariero L.H., Ohm I.K., Haugen F., Yndestad A., Solheim S., Seljeflot I., Ranheim T., Andersen G.Ø., Aukrust P. (2012). Increased circulating mitochondrial DNA after myocardial infarction. Int. J. Cardiol..

[38] Anguita E., Chaparro A., Candel F.J., Ramos-Acosta C., Martínez-Micaelo N., Amigó N., Torrejón M.J., Llopis-García G., del Mar Suárez-Cadenas M., Matesanz M. (2022). Biomarkers of stable and decompensated phases of heart failure with preserved ejection fraction. Int. J. Cardiol..

[39] Boonpala P., Saengklub N., Srikam S., Ji-Au W., Panyasing Y., Kumphune S., Kijtawornrat A. (2023). Pimobendan prevents cardiac dysfunction, mitigates cardiac mitochondrial dysfunction, and preserves myocyte ultrastructure in a rat model of mitral regurgitation. BMC Vet. Res..

[40] Oka T., Hikoso S., Yamaguchi O., Taneike M., Takeda T., Tamai T., Oyabu J., Murakawa T., Nakayama H., Nishida K. (2012). Mitochondrial DNA that escapes from autophagy causes inflammation and heart failure. Nature.

[41] Zhang Q., Raoof M., Chen Y., Sumi Y., Sursal T., Junger W., Brohi K., Itagaki K., Hauser C.J. (2010). Circulating mitochondrial DAMPs cause inflammatory responses to injury. Nature.

[42] Wang L., Zhang Q., Yuan K., Yuan J. (2021). mtDNA in the pathogenesis of cardiovascular diseases. Dis. Markers.

[43] Grigoriev E., Ponasenko A.V., Sinitskaya A.V., Ivkin A.A., Kornelyuk R.A. (2022). Mitochondrial DNA as a Candidate Marker of Multiple Organ Failure after Cardiac Surgery. Int. J. Mol. Sci..

[44] Verdejo H.E., Del Campo A., Troncoso R., Gutierrez T., Toro B., Quiroga C., Pedrozo Z., Munoz J.P., Garcia L., Castro P.F. (2012). Mitochondria, myocardial remodeling, and cardiovascular disease. Curr. Hypertens. Rep..

[45] Thassakorn P., Patchanee P., Pongkan W., Chattipakorn N., Boonyapakorn C. (2019). Effect of atorvastatin on oxidative stress and inflammation markers in myxomatous mitral valve disease in dogs: A comparison of subclinical and clinical stages. J. Vet. Pharmacol. Ther..

[46] Piantedosi D., Musco N., Palatucci A.T., Carriero F., Rubino V., Pizzo F., Nasir S., Molinaro G., Ruggiero G., Terrazzano G. (2022). Pro-Inflammatory and Immunological Profile of Dogs with Myxomatous Mitral Valve Disease. Vet. Sci..

[47] Reimann M.J., Häggström J., Møller J.E., Lykkesfeldt J., Falk T., Olsen L.H. (2017). Markers of Oxidative Stress in Dogs with Myxomatous Mitral Valve Disease are Influenced by Sex, Neuter Status, and Serum Cholesterol Concentration. J. Vet. Intern. Med..

[48] Verk B., Nemec Svete A., Salobir J., Rezar V., Domanjko Petrič A. (2017). Markers of oxidative stress in dogs with heart failure. J. Vet. Diagn. Investig..

[49] Zhan L., Wang X., Zhang Y., Zhu G., Ding Y., Chen X., Jiang W., Wu S. (2021). Benazepril hydrochloride protects against doxorubicin cardiotoxicity by regulating the PI3K/Akt pathway. Exp. Ther. Med..

[50] Tousoulis D., Papageorgiou N., Briasoulis A., Androulakis E., Charakida M., Tsiamis E., Stefanadis C. (2012). Conflicting effects of nitric oxide and oxidative stress in chronic heart failure: Potential therapeutic strategies. Heart Fail. Rev..

[51] Altoum A., Osman A.L., Babker A. (2018). Comparative study of levels of selective oxidative stress markers (malondialdehyde, zinc, and antioxidant vitamins A, E, and C) in ischemic and non-ischemic heart disease patients suffering from type-2 diabetes. Asian J. Pharm. Clin. Res..

[52] Romuk E., Wojciechowska C., Jacheć W., Nowak J., Niedziela J., Malinowska-Borowska J., Głogowska-Gruszka A., Birkner E., Rozentryt P. (2019). Comparison of Oxidative Stress Parameters in Heart Failure Patients Depending on Ischaemic or Nonischaemic Aetiology. Oxidative Med. Cell. Longev..

[53] Wojciechowska C., Romuk E., Tomasik A., Skrzep-Poloczek B., Nowalany-Kozielska E., Birkner E., Jacheć W. (2014). Oxidative stress markers and C-reactive protein are related to severity of heart failure in patients with dilated cardiomyopathy. Mediat. Inflamm..

[54] Romuk E., Wojciechowska C., Jacheć W., Zemła-Woszek A., Momot A., Buczkowska M., Rozentryt P. (2019). Malondialdehyde and uric acid as predictors of adverse outcome in patients with chronic heart failure. Oxidative Med. Cell. Longev..

[55] Bhat M.A., Gandhi G. (2019). Elevated oxidative DNA damage in patients with coronary artery disease and its association with oxidative stress biomarkers. Acta Cardiol..

[56] Qasim M., Bukhari S.A., Ghani M.J., Masoud M.S., Huma T., Arshad M., Haque A., Ibrahim Z., Javed S., Rajoka M.I. (2016). Relationship of oxidative stress with elevated level of DNA damage and homocysteine in cardiovascular disease patients. Pak J. Pharm. Sci..

